# Obituary Notice

**Published:** 1832

**Authors:** 


### ORIGINAL.

36. *Obituary.*—Died in this city on the 15th of October last, Dr. John E. Bush, for many years a highly respectable practitioner in this city.

Dr. Bush was a native of New-Jersey, in which state he completed his education, and was admitted to the practice of medicine in the spring of 1819. In the course of the ensuing summer he removed to this country and established himself in the practice of his profession in Brookville, Indiana, where, in a very short time, he acquired an unprecedented popularity and a very extensive practice.

Having been returned to the Legislature by the almost unanimous voice of his fellow citizens, in the session of 1822–3, he distinguished himself as an active, efficient, and influential member. Soon after, he removed to Cincinnati, where he formed an extensive acquaintance, and engaged in the practice of medicine with the most favorable prospects.

In 1827, Mrs. Bush was seized with a pulmonary affection which rapidly assumed the most alarming character, and induced the Doctor to relinquish his practice for the purpose of trying the effects of a southern climate. He left Cincinnati in the fall, with the intention of spending the winter in the West Indies, but the health of his wife declined so rapidly that they were compelled to remain in New-Orleans, where she fell a victim to her disease in March, 1828. He immediately returned to this place, where he continued to practice his Profession until his death.

On the evening of the 12th of October, soon after the appearance of the Cholera in this city, he was seized, after a day of excessive labor, with the premonitory symptoms of that disease. They were promptly arrested, however, and on the morning before his death he received the congratulations of his friends upon his fortunate escape. The symptoms, however, about the middle of the day, returning in the most aggravated form, he died in a few hours.

It was the happiness of the writer of this article to become acquainted with him while pursuing the elementary studies of his profession, and during an intimate acquaintance of years, he found him at all times exemplary in all the social and domestic virtues. He was a dutiful son, a tender and devoted husband, an affectionate parent, an ardent friend, and a public spirited citizen. Honest and upright in all his dealings, he abhorred every appearance of intrigue and unfair dealing in others not more than he avoided it himself. The friend of the poor—his readiness to relieve their distresses was only equalled by the kindness and sympathy with which his services were rendered.

As a member of the medical profession his pretensions were very respectable; his reading was extensive, and improved by a habit of original investigation. He was a member of the State Medical Society in 1828, and rendered valuable services in extablishing its present organization.

He was a man of easy manners, of varied powers of conversation, endowed with a lively and original vein of wit and humor, and of the most delicate sensibility. Fond of the study of nature and of the fine arts, especially the study of painting, he often amused himself with the pencil, in which he has left many mementoes behind him, happily conceived and neatly executed.

